# GAN: a platform of genomics and genetics analysis and application in *Nicotiana*

**DOI:** 10.1093/database/bay001

**Published:** 2018-02-21

**Authors:** Shuai Yang, Xingwei Zhang, Huayang Li, Yudong Chen, Long Yang

**Affiliations:** 1 Agricultural Big-Data Research Center and College of Plant Protection, Shandong Agricultural University, Taian 271018, China; 2 YuXi ZhongYan Tobacco Seed Co., LTD, Yuxi 653100, China; 3 Key Laboratory of Tobacco Pest Monitoring Controlling and Integrated Management, Tobacco Research Institute of Chinese Academy of Agricultural Sciences, Qingdao 266101, China

## Abstract

*Nicotiana* is an important Solanaceae genus, and plays a significant role in modern biological research. Massive *Nicotiana* biological data have emerged from in-depth genomics and genetics studies. From big data to big discovery, large-scale analysis and application with new platforms is critical. Based on data accumulation, a comprehensive platform of Genomics and Genetics Analysis and Application in *Nicotiana* (GAN) has been developed, and is publicly available at http://biodb.sdau.edu.cn/gan/. GAN consists of four main sections: (i) Sources, a total of 5267 germplasm lines, along with detailed descriptions of associated characteristics, are all available on the Germplasm page, which can be queried using eight different inquiry modes. Seven fully sequenced species with accompanying sequences and detailed genomic annotation are available on the Genomics page. (ii) Genetics, detailed descriptions of 10 genetic linkage maps, constructed by different parents, 2239 KEGG metabolic pathway maps and 209 945 gene families across all catalogued genes, along with two co-linearity maps combining *N. tabacum* with available tomato and potato linkage maps are available here. Furthermore, 3 963 119 genome-SSRs, 10 621 016 SNPs, 12 388 PIPs and 102 895 reverse transcription-polymerase chain reaction primers, are all available to be used and searched on the Markers page. (iii) Tools, the genome browser JBrowse and five useful online bioinformatics softwares, Blast, Primer3, SSR-detect, Nucl-Protein and E-PCR, are provided on the JBrowse and Tools pages. (iv) Auxiliary, all the datasets are shown on a Statistics page, and are available for download on a Download page. In addition, the user’s manual is provided on a Manual page in English and Chinese languages. GAN provides a user-friendly Web interface for searching, browsing and downloading the genomics and genetics datasets in *Nicotiana*. As far as we can ascertain, GAN is the most comprehensive source of bio-data available, and the most applicable resource for breeding, gene mapping, gene cloning, the study of the origin and evolution of polyploidy, and related studies in *Nicotiana*.

**Database URL**: http://biodb.sdau.edu.cn/gan/

## Introduction

The genus *Nicotiana* is the fourth largest genus in the family *Solanaceae*. Extensive research has accumulated a huge amount of data regarding genetics, evolution, genomics, taxonomy and breeding in the genus (1). Tobacco (*Nicotiana tabacum* L.) is cultivated worldwide as a plant of economic importance, as well as a model system in plant biotechnology (2). The collection and utilization of germplasm are the foundation of botanical research. A generally accepted international standard taxonomy for *Nicotiana* has existed for decades. It consists of 3 sub-genera, 14 sections and 66 species (3). However, most researchers now tend to classify *Nicotiana* into 13 sections and 76 species (4, 5). Regardless, several *Nicotiana* germplasm storehouses have been built over the years to collect and maintain *Nicotiana* germplasm resources. Presently, approximately 2152 *Nicotiana* accessions are maintained at the NPGS (National Plant Germplasm System, a world-famous genetic diversity warehouse for *N.tabacum*, http://www.ars-grin.gov/npgs/index.html) (6). In addition, a total of 1160 *Nicotiana* accessions are held at the CGRIS (Chinese Crop Germplasm Resources Information System, http://icgr.caas.net.cn/cgris_english.html) (7). Furthermore, the Tobacco Genetics and Breeding database (TGB, http://biodb.sdau.edu.cn/tgb/) also provides 1472 *Nicotiana* accessions with accompanying detailed annotations (8). Genus *Nicotiana* germplasm is generally understood to be available under >5000 accessions worldwide; however, no systematic database exists to preserve and catalogue all these germplasm resources.

The *N. tabacum* genome remained a challenge to sequence for quite a long time, because it is allotetraploid and very large. The allotetraploid (2*n* = 4x = 48) *N. tabacum* genome contains 24 pairs of chromosomes, derived from the two diploid species *N. sylvestris* and *N. tomentosiformis* (9, 10). High-throughput sequencing technologies have developed rapidly; comparative genomics can now provide insights into seven *Nicotiana* species: the allotetraploid *N. benthamiana* (11); three diploid species, *N. otophora*, *N. sylvestris* and *N. tomentosiformis* (10) and three common cultivated species, *N. tabacum BX*, *N. tabacum K326* and *N. tabacum TN90* (12). In the recent years, there was numerous sequences read archives available for other *Nicotiana* species like *N. obtusifolia*, *N. nudicaulis* and *N. repanda* and so on. Some of them were successfully used to identify allopolyploid species origin at a great accuracy (13, 14). So the high research value promoted to construct a genome information database which would provide convenient access to all the data.

The establishment of molecular markers and genetic linkage maps has greatly influenced gene mapping, gene cloning, quantitative trait loci and marker-assisted selection research (15, 16). As a model plant, genetic analyses in different *Nicotiana* genera employ a diversity of molecular markers (17–20). Molecular markers, restriction fragment length polymorphisms (RFLPs) (21), random amplified polymorphic DNAs (RAPDs) (22), amplified fragment length polymorphisms (AFLPs) (23), short sequence repeats (SSRs) (24), intron length polymorphisms (25), single nucleotide polymorphisms (SNPs) (26) and inter-simple sequence repeats (ISSRs) (27), were all excavated from previous research on a large scale. Ten *Nicotiana* genetic linkage maps were developed based on these markers, in our previous studies (28). However, these results were all derived from contig or scaffold sequences, not from whole genomes. Consequently, these marker linkage analyses needed to be redone on a large-scale using genome sequences.

An excellent resource for the online tobacco research community, the TGB database contains 1472 *Nicotiana* germplasms, with accompanying detailed annotations and 12 388 potential intron polymorphisms (PIPs), 10 551 EST-simple sequence repeats and 66 297 genomic-SSR markers (G-SSRs) (8). However, with the huge amount of new bio-data emerging in recent years, TGB can not satisfy all new research needs. Therefore, we found it necessary to establish a comprehensive platform of Genomics and Genetics Analysis and Application in *Nicotiana* (GAN, http://biodb.sdau.edu.cn/gan/). GAN contains a Source Section (Germplasm and Genomes), a Genetics Section (Markers, Genetics Maps and Genetics Annotations), a Tools Section [JBrowse (29) and Online Tools] and an Auxiliary Section (Datasets, Download and Manual) for *Nicotiana* research. GAN will greatly expedite genomics and genetics research in *Nicotiana* and related plants.

## Platform content and web interface

**Figure 1.**
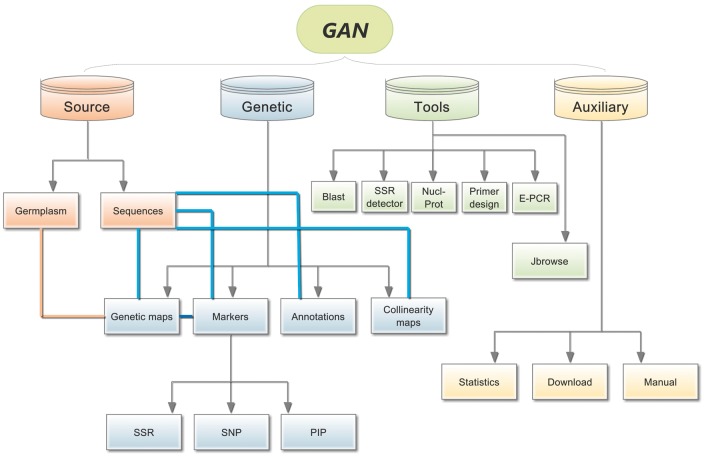
Overall GAN framework. The four different colours represent the four different main sections of GAN, Source, Genetics, Tools. Connections are illustrated between related subsections using straight lines with different colours.

GAN provides information regarding the detailed annotation of 5267 *Nicotiana* accessions, 7 genomes, 10 genetic linkage maps, 2239 KEGG metabolic pathway maps, 209 945 gene families, two co-linearity maps, 3 963 119 genome-SSRs, 10 621 016 SNPs, 12 388 PIPs, 102 895 reverse transcription-polymerase chain reaction (RT-PCR) primers, JBrowse and 5 useful online softwares. The GAN Web interface was designed to include the following components: Source, Genetics, Tools and Auxiliary (Figure 1).

JBrowse and the five online bioinformatics tools, Blast (30), Primer3 (31), SSR-detect (32), Nucl-Protein and E-PCR (33), are provided on the JBrowse and Tools pages. An Auxiliary Section contains all the datasets in GAN, displayed on a Statistics page and available for download on the Download page. In addition, the Auxiliary Section contains a user’s manual, provided on the Manual page in English and Chinese languages. GAN provides a user-friendly Web interface for searching, browsing and downloading genomics and genetics datasets for *Nicotiana*. As far as we know, GAN provides the most comprehensive *Nicotiana* bio-data available, and is an excellent resource for research in *Nicotiana* breeding, gene mapping, gene cloning, the origin and evolution of polyploidy and related studies.

**Figure 2.**
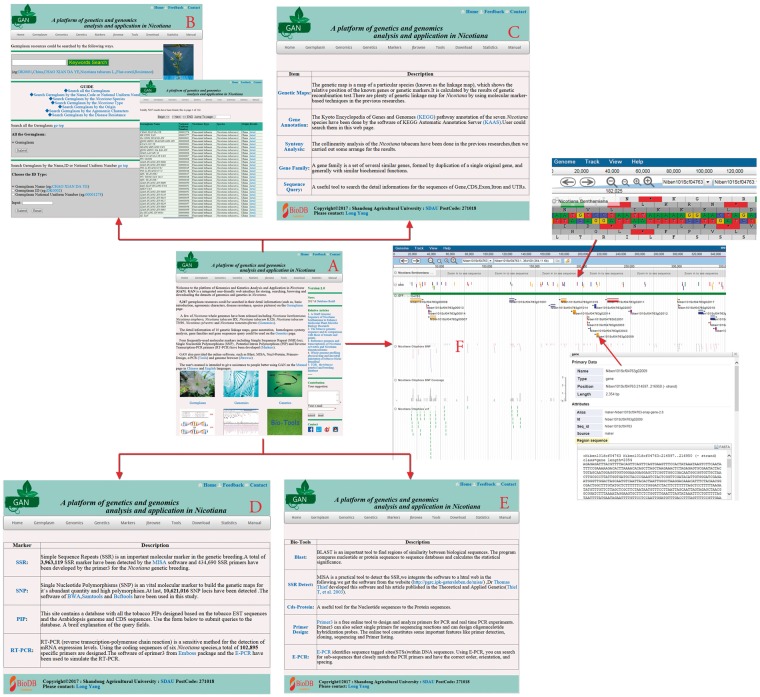
Main GAN Web page. (**A**) GAN homepage: provides quick entry paths to all main parts. (**B**) Germplasm page: stores 5267 *Nicotiana* accessions, detailed information and a seven-mode search mechanism. (**C**) Genetics page: consists of genetic maps, gene annotations, synteny analyses, gene family identifications and sequence query. (**D**) Markers page: includes all SSR, SNP, PIP and RT-PCR markers in GAN. (**E** and **F**) Tools and JBrowse pages: allow access to online bioinformatics tools and the genome browser JBrowse for detailed information overlays and analyses.

The GAN platform provides researchers all necessary data, and is composed of five main parts: Germplasm, Genomics, Genetics, Markers and Tools (Figure 2). (i) The Germplasm Section holds 5267 *Nicotiana* accessions, which can be queried using several different routes: specific ID, species, *Nicotiana* type, cultivar origin, agronomic characteristics and disease resistance characteristics. Furthermore, detailed germplasm information is also provided with high-quality images from the field or laboratory. (ii) The Genomics Section contains the seven available *Nicotiana* genome sequences, and all sources and annotations. (iii) The Genetics Section contains genetic maps, gene annotations, synteny analyses and gene family make up analyses for those seven *Nicotiana* species with complete genome data. (iv) The Markers Section contains all of the three widely used molecular markers, SSRs, SNPs and PIPs, that have been detected in those seven *Nicotiana* genes, and some of the exons, introns and UTRs. Furthermore, because RT-PCR is such a sensitive and widely used method for the detection of mRNA expression levels, RT-PCR primers are also available, predesigned using the coding sequences (CDSs) of current *Nicotiana* species. (v) The Tools Section contains the online bioinformatics tools Blast, SSR-detect, Primer3, Nucl-Protein and E-PCR, which have all been integrated into GAN, as well as the currently popular genome browser JBrowse for the display of detailed annotation and marker information overlaid on the genome.

### Germplasm

**Figure 3.**
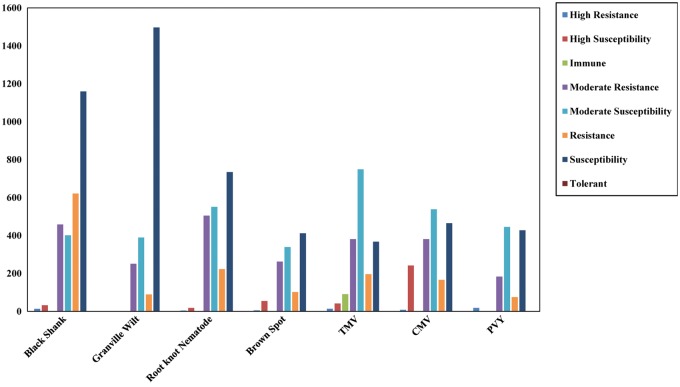
Detailed disease resistance information for the GAN *Nicotiana* accessions.

We divide the 5267 GAN accessions into 35 *Nicotiana* species, of which ∼92.8% are *N. tabacum* L., in accordance with accepted *Nicotiana* classification. The germplasm type is differentiated into seven tobacco categories: Aromatic, Burly, Cigar, Flue-cured, Wild, Rustica and Sun-cured. The 5267 *Nicotiana* accessions come from 45 different cultivar origins, with most cultivar originating in China and the next most from North America (Table 1). Detailed disease resistance information is provided for all accessions, including Black Shank, Granville Wilt, Root-knot Nematode, Brown Spot, Tobacco mosaic virus, Cucumber mosaic virus and Potato virus Y (Figure 3).

**Table 1.** Distribution of 5267 *Nicotiana* accessions

Cultivar Origin	Aromatic tobacco	Burley tobacco	Cigar tobacco	Flue-cured tobacco	Wild tobacco	Rustica tobacco	Sun-curing tobacco	Total
China	17	90	5	1762	0	328	2270	4472
America	0	64	13	287	4	5	27	400
Zimbabwe	3	3	0	17	0	1	0	24
Poland	4	4	6	5	0	0	2	21
Yugoslavia	8	0	3	4	0	0	2	17
Japan	6	4	0	13	1	3	4	31
Bulgaria	14	0	0	0	0	0	0	14
Canada	0	0	0	12	0	0	1	13
Australia	0	0	0	3	6	0	1	10
Others	43	35	28	100	24	7	28	265
Total	95	200	55	2203	35	344	2335	5267

The Germplasm page can be searched using ID-Name, species, type, cultivar origin, agronomic characteristics and disease resistance keywords. Inputting your desired words and clicking the ‘Keywords Search’ button initiates a global keyword search. Users can also click the ‘Submit’ button in the Germplasm gray-box to browse all germplasms, displaying all 35 *Nicotiana* species, 7 *Nicotiana* types and 45 cultivar origins, which can then be chosen among. Finally, three different specific identifiers, the germplasm’s name, its GAN ID and its National Uniform Number, can be used to search for a specific germplasm. Thirty-five common field agronomic characteristics are provided including phenotype and physiology. Users can choose one or more parameters to generate exact results. Similar to agronomic characteristics, the disease resistance search box offers seven kinds of disease, including different levels of resistance, such that the user can obtain accurate germplasm identifications. Results in the Web interface appear as simple information entries in tables in which users can click a ‘Detail’ hyperlink to generate detailed information about a specific germplasm. The Detail Web page displays four main attributes: basic information, agronomic characteristics, disease resistance and *Nicotiana* images for each germplasm. The basic information entry provides the *Nicotiana* species of the germplasm along with an introduction, common names, economic importance and references.

### Genomics

Seven sequenced *Nicotiana* genomes, including assembly and annotation information, genome size (∼2.7 G through ∼5.18 G), contigs and scaffolds can be found on the Genomics page.

### Genetics

Ten specific genetic maps were constructed in previous research using different RFLP, RAPD, ISSR and SSR molecular markers. Wild species, Burly and Flue-cured are all used as materials among the 24 chromosomes. Enzymes of 2115 relate to metabolic pathways found in 6 *Nicotiana* CDSs, which participate in 383 specific pathway maps. A total of 506 553 protein domains and/or families were identified using Pfam, Pkinase, WD domain and PPR family analyses in the 6 *Nicotiana* CDSs.

Users can browse ten genetics maps in the Genetics Section. Tables store constitutive data for these genetic maps in the main Web page. A graphical ‘Go’ button is pressed to get the requested genetic map; the maps contain detailed marker information obtained by clicking on the marker. KEGG annotation can then be queried if users select a specific *Nicotiana* species and input a sequence ID or KEGG Orthology number. Gene family, *Nicotiana* species or type, Gene ID, PF(Plant Family) accession and name can all be used to start the search.

### Molecular markers

A total of 3 429 801 G-SSRs, 326 253 gene-SSRs and 18 485 CDS-SSRs were catalogued in this research. Additionally, 267 699 primer pairs were designed for *Nicotiana* genes. SNP markers of 10 621 016 were searched against and located on the reference *N. benthamiana* genome (Table 2). Furthermore, 12 338 PIP makers were obtained from the TGB platform. Finally, 102 859 pairs of E-PCR verified primers were generated for scientific research.

**Table 2.** The statistics of SSRs and SNPs

Species	Genome-SSR	Gene-SSR	SNP
*Nicotiana benthamiana*	492 540	61 568	-
*Nicotiana otophora*	358 420	-	785 204
*Nicotiana sylvestris*	420 177	63 159	1 872 257
*Nicotiana tabacum BX*	594 364	47 536	2 391 165
*Nicotiana tabacum K326*	620 818	45 437	2 403 132
*Nicotiana tabacum TN90*	637 331	44 660	2 442 813
*Nicotiana tomentosiformis*	306 151	63 893	726 445

Generally, all of the Markers search pages require a *Nicotiana* species to be chosen as a necessary option. Different markers have their own search options beyond that: The SSR page provides options for sequence type, Gene ID, SSR motif and repeat number; the SNP page allows for searching by genome sequence ID, reference base and query base; the PIP page contains options for PIP ID, PlantGDB ID(http://www.plantgdb.org/) and gene name; the RT-PCR page requires a gene sequence ID. Clicking the ‘Detail’ hyperlink in the Result Web interface for all markers displays detailed information on the marker.

### Tools

Five frequently used software tools are provided in GAN (Blast, Primer3, SSR-detect, Nucl-Protein and E-PCR) and one genome browser (JBrowse). Generally speaking users can paste sequences in FastA format directly into a text-area, or upload sequence files from a local computer, into the five software pages. Users select from five Blast variants (blastp, blastx, blastn, tblastx and tblastn) on the Blast page, and search against any of the seven *Nicotiana* species genome sequences (or corresponding translations) along with basic program parameters to obtain results. In the SSR-detector page, SSRs can be identified using the default parameters of MISA (34). The Nucl-Protein page provides a tool for the translation of nucleotide sequences to protein sequences; users only need to provide a nucleotide sequence and working name, similarly with SSR-detector. The Primer Design page requires users to input the Min-TM, Max-TM, Min-GC, Max-GC and return number to predict maximal Primer3 results. The primer verification tool E-PCR uses the Primer3 results and requires users to select one *Nicotiana* species as a template, followed by a few alternative parameters. Results are obtained by online perusal or can be download to the local computer.

JBrowse is an open-access genome browser that allows for the storage and display of genome sequences, genes, mRNAs, CDSs, introns, exons, UTRs, SSRs and SNPs, providing detailed annotation for each. In our implementation, the left column lists options consisting of *Nicotiana* genomes, a GFF choice, SNP loci, SNP coverage and SSR locations. The right column will display the corresponding information when users click the square box before the options in the left column. The GFF option provides an example showing how to work with the bowser. By clicking the GFF square all genome information will graphically display, then a dialog box will pop up if you click a specific graphic. Users can browse and download detailed information from the dialog box. We have added several new and useful features to the basic JBrowse browser (Figure 2).

### Download and help

Users can download all the data used in GAN on the Download page. A download tree is used to access all the data relative to the Germplasm, Genomics, Genetics and Markers pages.

## Conclusions and perspectives

GAN is a platform for providing *Nicotiana* accessions while integrating useful genomic and genetic data, and supplying relevant annotations and molecular markers in *Nicotiana*. The 5267 *Nicotiana* accessions are the main content of the platform; however, GAN also expedites scientific work by identifying *Nicotiana* gene annotations and markers and primers for genetics analysis and breeding. Furthermore, the incorporation of several useful tools and JBrowse make it easy for users unfamiliar with bioinformatics to perform big-data scientific research. The platform will be updated continuously as new *Nicotiana* accessions are collected, and new *Nicotiana* genome sequences are generated.

## Materials and methods

### Germplasm and sequences

Information on 51 traits, and plant and inflorescence images of 5267 *Nicotiana* accessions were exhaustively collected. The genome sequences of *N. benthamiana*, *N. otophora*, *N. tabacum* BX, *N. tabacum* K326 and *N. tabacum* TN90 were downloaded from SGN (35) (Sol Genomics Network, https://solgenomics.net/) and the genome sequences of *N. sylvestris* and *N. tomentosiformis* were obtained from NCBI (National Center for Biotechnology Information, https://www.ncbi.nlm.nih.gov/).

### Genetic maps and annotation

Ten *Nicotiana* genetic linkage maps with associated molecular markers were collected from previous research. Co-linearity maps with tomato and potato were also extracted from previous *Nicotiana* sequence research (12). All relevant information can be found on the appropriate GAN page.

The KAAS Web interface (36) (http://www.genome.jp/tools/kaas/) was used annotate data with the KEGG pathway assignments (37) (Kyoto Encyclopedia of Genes and Genomes). PfamScan software (38) (http://www.ebi.ac.uk/Tools/pfa/pfamscan/) was used to scan gene families from Pfam (39) (http://pfam.xfam.org/).

### Marker identification

From different type sequences of *Nicotiana* species, the SSRs (Genome-SSR, Gene-SSR, Cds-SSR, Exon-SSR and Intron-SSR) were detected by scanning monomer, dimer, trimer, tetramer, pentamer and hexamer nucleotide motifs with at least 10, 6, 5, 5, 5 and 5 repeats by using perl-based MISA program (http://pgrc.ipk-gatersleben.de/misa/), respectively. For complex SSRs, the maximum difference between two SSRs had to be <100 bp. Primer3 was used for SSR primers design (31). Using *N. benthamiana* sequences as reference, and the other six *Nicotiana* species sequences as queries, SNPs were extracted and filtered using the BWA (Burrows-Wheeler Aligner) (40), Samtools (41) and Bcftools. Among them, BWA is responsible for database building and comparison. Samtools is responsible for processing and converting formats. Finaly, Bcftools was used for SNP calling. All parameters were used at default setings.

### Platform implementation

The LAMP framework (Linux, Apache, MySQL and PHP/Perl) was used to construct the GAN platform. All standardized data were imported into MySQL for storage and management. Commands are submitted in the Web-friendly interface using HTML on the Apache Web server, generating PHP scripts, which extract corresponding data from MySQL. Perl and Java scripts are used to enhance the appearance of the Web interface and to improve user query efficiency.
